# COVID‐19, when fourteen days are not enough—A case series of affected healthcare workers

**DOI:** 10.1002/ccr3.3705

**Published:** 2021-03-13

**Authors:** Catherine Murphy, Noirin Noonan, Eilis O’Toole, Patrick Plunkett, Mary Paula Colgan, Caitriona Canning, Zenia Martin, Martina Hennessy

**Affiliations:** ^1^ St James Hospital Dublin Ireland

**Keywords:** COVID‐19, healthcare worker, prolonged course, rehabilitation program, return to work

## Abstract

We highlight the need for planning for mass workforce absentees as we prepare for subsequent surges. We suggest a multicomponent intervention including guiding return dates more by symptomatology and fitness for work rather than infectivity status.

## Graphical abstract

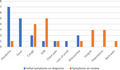

## 1 INTRODUCTION

This case series identified a subcohort of healthcare workers with COVID‐19 who experienced a prolonged symptomatic course and remained unable to return to work for many weeks. It notes their demographic and clinical characteristics and suggests ways to support them returning to work in a graded and supported manner.

Coronaviruses are known pathogenic microorganisms in both humans and animals.^1^

Four of the seven known coronaviruses that cause disease in humans only cause mild‐to‐moderate flu‐like illnesses.^1^ Three are known to cause more serious disease, including fatality. SARS coronavirus (SARS‐CoV) emerged in November 2002 causing severe acute respiratory syndrome (SARS).^1^ Middle East respiratory syndrome (MERS) is caused by the MERS coronavirus (MERS‐CoV).^1^ It was identified in September 2012 and continues to cause sporadic and localized outbreaks.^1^ Severe acute respiratory syndrome coronavirus 2 (SARS‐CoV‐2) was identified as the cause of a cluster of pneumonia cases in Wuhan, a city in the Hubei Province of China in December 2019.^2^ Since then, it has spread internationally to 216 countries causing more than 340 000 deaths as of 25 May 2020.^2^ It continues to threaten healthcare workers globally. Since its first arrival in Wuhan in December 2019, the idiosyncratic nature of the SARS‐CoV‐2 infection has sparked both interest and concern among healthcare workers globally.

An early study of hospitalized patients from a single institution in Wuhan showed that on admission, most patients had fever or cough, and a third of patients had shortness of breath. Other symptoms included muscle ache, headache, confusion, chest pain, and diarrhea. Several patients proceeded to get organ function damage, including 17 (17%) with ARDS, eight (8%) with acute respiratory injury, three (3%) with acute renal injury, four (4%) with septic shock, and one (1%) with ventilator‐associated pneumonia.^3^ Venous thromboembolism is common in patients with severe COVID‐19 infection.^4^ Studies advise initial investigation with CBC, PT, aPTT, fibrinogen, and D‐dimer. The results of these markers have also been shown to correlate with disease severity and mortality.^4^ A larger study of 1099 patients with confirmed COVID‐19 infection from 552 hospitals in China showed the virus had a median incubation period of 4 days. The median age was 47. Fever was detected in 43.8% of the patients on admission but developed in 88.7% during hospitalization. The second most common symptom was cough (67.8%). Among the overall population, 23.7% had at least one coexisting illness.^4^ There were 926 patients who were categorized as nonsevere on admission and 173 who fell into the severe category.^5^ Patients with severe disease were older than those with nonsevere disease by a median of 7 years.^5^

Our knowledge of COVID‐19 presentations is still evolving; there have been many case reports of atypical presentations of COVID‐19 infection including one case report suggesting altered mental status as a presenting complaint for an elderly patient.^6^ The World Health Organization report that 80 percent of infections “are mild or asymptomatic.”^2^ Despite these seemingly “mild” cases, many patients seem to suffer with a prolonged course and fail to return to their pre‐COVID baseline for some time. Online support groups have thousands of people who continue to grapple with the effects of COVID‐19 infection months after the onset of symptoms calling themselves “long‐termers” or “long haulers.”

Other coronaviruses, SARS‐CoV and MERS‐CoV, have been associated with rapid spread in healthcare settings often resulting in large nosocomial outbreaks. Hospital overcrowding, workplace absenteeism, nonadherence to infection prevention and control measures, and possible environmental contamination are all thought to be implicated in such amplification in the case of MERS‐CoV outbreaks.^7^, ^8^, ^9^, ^10^, ^11^

Our understanding of COVID‐19 infection among health workers and those at risk of adverse outcomes and prolonged courses is important for our understanding of virus transmission patterns and for preventing the future infection of health workers and other patients. A better understanding of the workforce absentees and difficulties with returning to work will allow us to plan for appropriate staffing and support for our healthcare workers. It is also crucial for informing and updating infection prevention and control measures at healthcare facility and national level, and for reducing secondary COVID‐19 transmission within healthcare settings. Ireland faces one of the highest rates of diagnosed healthcare worker infection in Europe. The European Centre for Disease Prevention and Control found on 8 April that across Europe, “between 9% and 26% of all diagnosed COVID‐19 cases are in healthcare workers.” In Ireland, 8018 healthcare workers were diagnosed with COVID‐19 of which 4823 remain sick. 1600 of these were nurses and midwives.^12^ The figures show that healthcare workers make up over a quarter of the COVID‐19–positive cases tested in Ireland.

In this case series, we identified a cohort of health workers with a laboratory‐confirmed COVID‐19 infection who experienced a prolonged symptomatic course of COVID‐19 infection beyond 21 days.

### 1.1 Objectives

To describe and identify the clinical characteristics of healthcare workers who experienced a prolonged symptomatic phase of COVID‐19 infection and required a more substantial recovery phase greater than fourteen days which was deemed the end of infectivity.

## 2 CASES—HISTORY AND EXAMINATION

The hospital is a 781‐bedded tertiary care center in inner Dublin, Ireland, with over 5000 staff. Healthcare workers with suspected COVID‐19 infection on the basis of initial symptoms were referred for swabbing by the Occupational Health Department. If deemed positive, they were contacted by phone by the Infectious Diseases team to enable clinical care if they deteriorated and by Occupational Health, to discuss procedures for return‐to‐work evaluation. A direct line to Occupational Health was given to healthcare workers for contact if concerned about their symptoms. Contact was also made by Occupational Health to healthcare workers as they approached their return‐to‐work date to assess if suitable to return to workplace duties. When contact led to concern by the Occupational Health physicians, patients were referred for clinical review. Depending on the urgency of their clinical state as evaluated by telephone, this was either to the emergency department for immediate assessment or referral to a dedicated clinic setup for COVID‐positive healthcare workers. Our patients were recruited from the COVID‐positive healthcare worker clinic. Informed consent was obtained from all patients, and this study was approved by St. James and Tallaght Hospital Joint Research Ethics Committee.

A confirmed case of COVID‐19 was defined by a positive result on a reverse transcriptase polymerase chain reaction (RT‐PCR) assay of a specimen collected on a nasopharyngeal swab. Only laboratory‐confirmed cases were included. The assay used to confirm COVID‐19 infection is based on the World Health Organization standard and was confirmed by RT‐PCR in the routine laboratory using either MutaPLEX (Immundiagnostik AG) or RealTime SARS‐CoV‐2 (Abbott).

From a total of 456 healthcare workers with laboratory‐confirmed COVID‐19 infection in our institution, a total of 203 (44.5%) remained off work for greater than 21 days. Of these, we reviewed 19 healthcare workers in our clinic who were experiencing a prolonged symptomatic phase > 21 days from initial symptom onset and whose symptoms were deemed significant enough to require medical review and thus referred to our COVID‐19 healthcare worker clinic. We excluded healthcare workers who had been admitted with their symptoms. The longest period of symptoms since onset in our cohort was 57 days, and the shortest period was 27 days. The demographic and clinical characteristics of the patients are shown below. The majority of patients were female at 80% of the cohort (Table 1). In our institution, 79% of our healthcare workers are female with 89% of nursing staff being female and 61% of healthcare assistants. The mean age of the patients was 41.68 yrs. The most common occupation within the healthcare profession was nursing (52.6%) (Table 1). All patients were nonsmokers, 76.4% had never smoked with the remaining 23.6% were ex‐smokers. Thirty‐two percent had no medical comorbidities (Table 1). The most common comorbid conditions were asthma (15.6%) and hay fever (15.6%) (Table 1).

**TABLE 1:** Baseline characteristics

		Column N%
Gender	Male	20%
	Female	80%
Comorbidities	Hypothyroid	5.3%
	GORD	5.3%
	Irritable bowel syndrome	5.3%
	Migraine	5.3%
	Obesity	5.3%
	Type 1 diabetes	5.3%
	Overactive bladder	5.3%
	Asthma	15.8%
	Hay fever	15.8%
	No medical comorbidities	31.6%
Ethnicity	Polish	5.3%
	Chinese	5.3%
	Mauritian	5.3%
	African American	10.5%
	Filipino	10.5%
	Indian	15.8%
	Irish	47.4%
Occupation	Phlebotomist	5.3%
	Catering	10.5%
	Healthcare assistant	31.6%
	Nurse	52.6%

**FIGURE 1:**
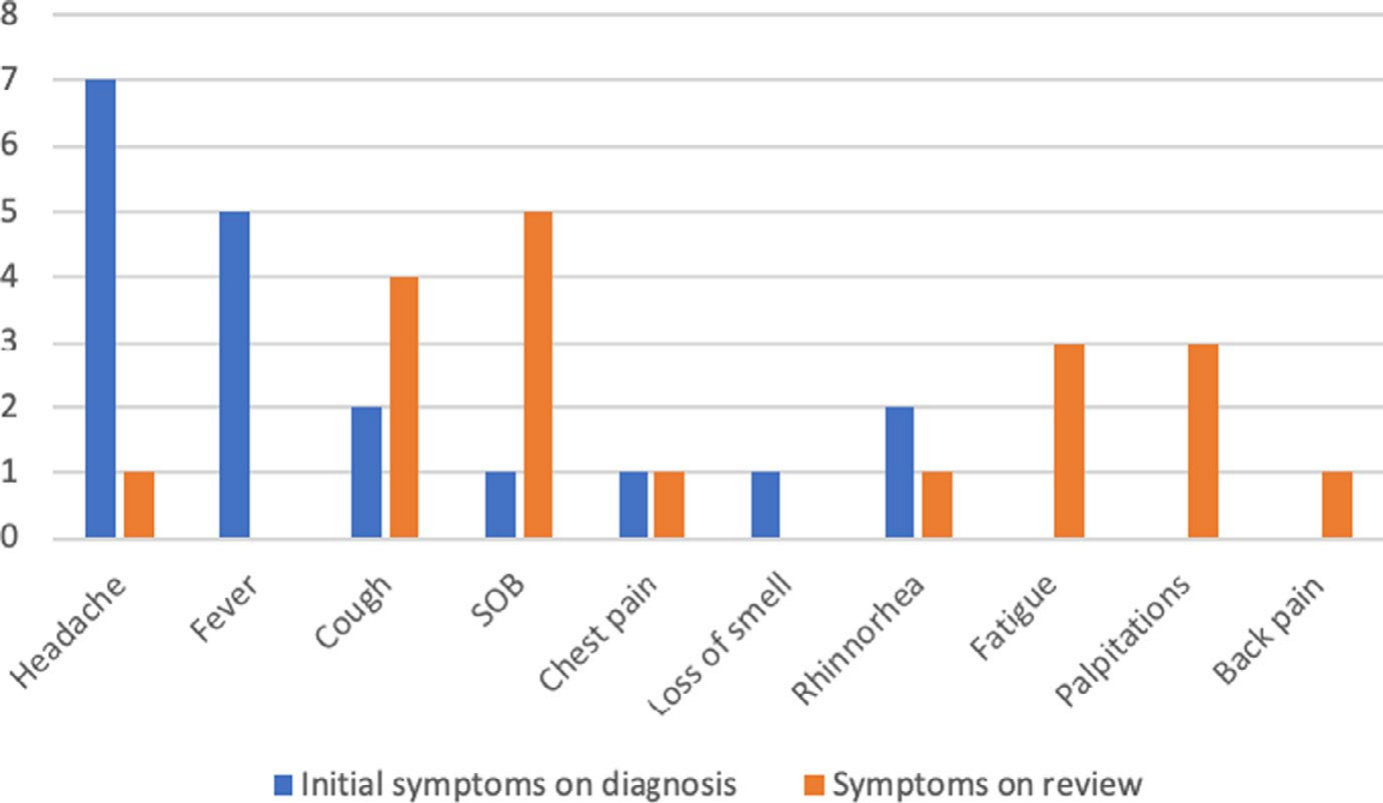
Bar chart—symptoms at initial diagnosis vs symptoms at the time of review. †—figure represents each patient's main symptom only

The mean (±SD) number of days since positive swab until review in clinic was 36.47 (12.043) with a median of 32 (Table 2). The mean (±SD) number of days since symptom onset was 39.63 (11.767) with a median of 35 (Table 2). The most common initial symptom at the time of diagnosis was headache (36.8%), followed by fever (26.3%) (Figure 1). However, there was a shift in symptomatology between initial diagnosis and time of review in clinic. The most common symptom at presentation to the review clinic was shortness of breath, (25%) followed by cough (21%), palpitations (15.7%), and fatigue (15.7%) (Figure 1).

**TABLE 2:** Time from symptoms and positive swab

	Days since swab	Days since symptoms
Mean	36.47	39.63
Median	32.00	35.00
SD	12.04	11.77

On clinical examination, the mean heart rate at rest was 86 beats per minute and the mean oxygen saturation was 98% on room air. Three patients who presented with palpitations had a resting sinus tachycardia > 100 bpm. On evaluation of a six‐minute walk test, 10.5% of patients seen desaturated below 94% when mobilizing.

## 3 INVESTIGATIONS

The table shows the mean laboratory values of patients on review in the outpatient clinic. Most patients had normal inflammatory markers at this stage in the course of their illnesses. Only four patients (21%) were lymphopenic at the time of review (Table 3). Most patients had normal chest X‐rays (CXR), with only 15.7% having infiltrates on CXR. Two patients had significantly elevated D‐dimer values who then progressed to have CT pulmonary angiograms where no pulmonary embolisms were identified (Table 3). Three patients proceeded to have echocardiograms which demonstrated normal ejection fractions and no evidence of cardiomyopathy. Two patients had Pulmonary function tests, one of which had a mildly reduced FVC at 72% predicted and the other had pulmonary function tests, which were within normal range.

**TABLE 3:** Laboratory values

	Normal values	N	Minimum	Maximum	Mean	SD
Lymphocytes(10^9^/L)	1.5‐3.5	19	1.0	3.0	1.968	0.5784
White blood cells (10^9^/L)	4‐10/nL	19	2.9	11.4	6.095	1.9845
C‐reactive protein (mg/L)	<5 mg/l	19	0	5.47	1.25895	1.922169
Interleukin‐6 (pg/ml)	0.09‐ 7.26	13	<3.130	4.1	3.21846	0.266422
D‐dimer (ng/mL)	<500 ng/ml	15	<215	7266	795.73	1794.402
Ferritin (µg/L)	23.0‐393.0	18	7.1	437.0	124.961	130.1876
25‐OH vitamin D (nmol/L)	>50	13	19	92	47.92	21.418

## 4 DISCUSSION

This study describes 19 healthcare workers treated as outpatients with confirmed COVID‐19 infection who experienced a prolonged symptomatic phase and remained unfit to return to work greater than twenty‐one days from confirmation of diagnosis. This cohort represents a 4.17% subgroup of healthcare workers at our institution who were infected with COVID‐19. An evolution in symptomatology was observed from initial presentation with fever and headaches to respiratory symptoms and fatigue in the more chronic stage.

The number of infected and exposed healthcare workers who remain unfit for workplaces an immense strain on an already pressurized healthcare system. As is the case in this cohort, many infected healthcare workers cannot return to work for prolonged periods due to persistence of COVID‐19 infection symptoms. Given that returning to work for a large proportion of healthcare workers involves long shift work hours often involving 12‐ to 13‐hour shifts, we must recognize and identify these patients who are experiencing a prolonged course and support their return to work in a graded fashion once they have recovered from their illnesses.

When we look to other viral illnesses and their effect on the workforce, we can recognize the significance such prolonged courses may have. A study in the Journal of Occupational Health published in 1998 reviewed the impact of influenza and influenza‐like illness on healthcare resource utilization. Patients were incapacitated or confined to bed for 2.4 days, leading to workplace absenteeism of 2.8 days per episode of illness.^13^ On return to work, they reported reduced effectiveness and inability to resume normal activity until a mean of 3.5 days after the onset of symptoms. Each participant reported a mean of 6.5 days of influenza or influenza‐like symptoms.^13^ There was a positive correlation between the number of symptoms and bed days and missed work days.^13^ These figures bring to light an alarming reality in the face of the global COVID‐19 pandemic. Given that this study reports healthcare workers experiencing much more severe and debilitating symptoms with COVID‐19 infection and a more prolonged course, it follows that we can expect to see a prolonged reduction in workplace effectiveness and a longer duration until staff are able to resume their normal duties.

If we look to previous experience with coronaviruses, longer‐term follow‐up studies have shown that patients with severe acute respiratory syndrome (SARS‐CoV‐1) may experience long‐term symptoms, such as restrictive pulmonary dysfunction, palpitations, hand tremors, and exertional dyspnea, all of which have an effect on their ability to return to their premorbid life and daily activities.^14^ It has been suggested that these symptoms are associated with prolonged bed rest, adverse effects of steroid medications, and residual pathological changes, such as atelectasis, persistent alveolitis, pulmonary fibrosis, and varying degrees of muscle weakness or dysfunction.^14^ Given that many patients with COVID‐19 infection would have spent prolonged periods in bed and were often confined to a single room on isolation for a minimum of fourteen days, it is reasonable to expect an element of deconditioning and weakness on return to normal function.

Furthermore, data are beginning to emerge on the longer‐term lung damage that can be caused by COVID‐19 infection. A study by Wang et al looked at 70 patients who had survived COVID‐19 pneumonia in Wuhan China and found that 94% of discharged patients had evidence of residual disease on final CT scans, with ground‐glass opacity being the most common pattern of disease.^15^ An increasing awareness of the post–COVID‐19 lung is beginning to emerge in the literature for which respiratory rehabilitation may be helpful, but there is a danger this population of healthcare workers may be overlooked because of their seemingly mild cases at initial presentation. Similarly in SARS and MERS, many patients experienced long‐term respiratory dysfunction. When we consider SARS, a study looking at long‐term lung damage in 71 affected patients showed that at 15 years, 4.6% (SD 6.4%) of the lungs showed interstitial abnormality in patients who had been infected.^16^ Comparatively, CT abnormalities in patients infected with MERS included bilateral ground‐glass opacities, most commonly in the basal and peripheral areas.^17^ Although follow‐up data are less well described in MERS, one study of 36 patients looked at chest X‐rays taken a median of 43 (range: 32‐320) days postdischarge from hospital and showed abnormalities described as lung fibrosis in about a third of the patients.^17^ The suggestion that we may witness similar long‐term outcomes from patients infected with COVID‐19 infers that we should prepare to support our healthcare settings with sufficient staffing levels to allow for potential absenteeism and support our workers to face the possibility of longer‐term disability and adjust rostering and workplace duties accordingly.

Along with the risks of direct infection which arises from working in close proximity with COVID‐19–confirmed cases and with potentially infectious colleagues, healthcare workers are also under increasing stress and mental health risks. Observations were made by assessors of emotional lability of patients during assessment; however, due to inconsistent documentation of psychological state we were unable to include this in the analysis. It was noted that patients were often tearful during consultation and reported feelings of mental and social isolation. Comparatively, patients with SARS and MERS, in the recovery phase of the illness, experienced sleep disorder, frequent recall of traumatic memories, emotional lability, impaired concentration, fatigue, and impaired memory in more than 15% at a follow‐up period between 6 weeks and 39 months.^18^ When interpreting these data with respect to COVID‐19 infection, we must be cautious as very little data on the post–illness phase exist; however, it might be reasonable to expect a more significant psychological impact due to the higher mortality and more severe clinical course. When we consider this population of healthcare workers, many of whom were involved in caring for acutely unwell infected patients feelings of emotional lability and recall of traumatic memories can be expected. Longer‐term data from SARS and MERS indicate that the prevalence of depression, anxiety, post‐traumatic stress disorder, and fatigue from COVID‐19 infection should be expected to be high.^18^

This study has several limitations. The study population only included those healthcare workers who presented as they failed to meet their return‐to‐work date or whose ID was concerned enough about to refer for medical review. Many healthcare workers may have returned to work and yet may still continue to feel symptomatic. Another limitation is that the data were collected at a single time point from the electronic health record database; therefore, the level of detail was limited to documentation on initial consultation.

Further research is needed to inform our recognition of healthcare workers who are experiencing prolonged symptomatic courses of COVID‐19 infection and to support these patients in achieving a full recovery and a return to the workforce. Consideration should be made for formalized long‐term follow‐up to ensure a return to full health or further medical support as required. Return‐to‐work dates should be guided more by patient symptomatology and fitness for work rather than ineffectively status. Healthcare workers should be given the opportunity to return to work in a more gradual manner, and a formal follow‐up should be arranged to ensure they are re‐adjusting back to both the physical and mental challenges of returning to duties in the COVID‐19 pandemic. Further research is needed to assess the role of rehabilitation programs for healthcare workers to assist them with returning to their normal duties and assessment of their workplace effectiveness on return to work. Our findings also highlight the need for planning for mass workforce absentees as we deal with the consequences of the COVID‐19 pandemic and prepare for subsequent surges.

## CONFLICT OF INTEREST

None declared.

## AUTHOR CONTRIBUTIONS

Catherine Murphy and Professor Hennessy: conceived the study. Noorin Noonan, Eilis O’Toole, Professor Plunkett, Mary Paula Colgan, Catrionia Canning, and Zenia Martin: initiated the study design and helped with implementation. Catherine Murphy: did the statistical analysis and prepared the manuscript. All authors: contributed to the refinement of the study protocol and approved the final manuscript.

## ETHICS STATEMENT

This study was ethically approved by the Joint James Tallaght Ethics Committee. JREC reference: 2020‐05 list 19.

## Data Availability

All available data can be obtained by contacting the corresponding author.
